# Significant Cytokine mRNA Expression Changes Immediately after Initiation of Cardiopulmonary Resuscitation

**DOI:** 10.1155/2017/8473171

**Published:** 2017-12-27

**Authors:** Mareen Braunstein, Martina Williamson, Thomas Kusmenkov, Jürgen Landes, Peter Biberthaler, Karl-Georg Kanz, Wolfgang Böcker, Viktoria Bogner

**Affiliations:** ^1^Department of Trauma Surgery, University Hospital Munich, Ludwig Maximilians University, Nussbaumstr. 20, 80336 Munich, Germany; ^2^Department of Trauma Surgery, Chirurgisches Klinikum München Süd, Am Isarkanal 30, 81379 Munich, Germany; ^3^Department of Trauma Surgery, Klinikum Rechts der Isar, Technical University of Munich, Ismaninger Strasse 22, 81675 Munich, Germany

## Abstract

**Introduction:**

The purpose of this study was to evaluate immediate immunological changes following cardiopulmonary resuscitation (CPR). mRNA expression levels of selected immunomodulatory cytokines in out-of-hospital cardiac arrest (OHCA) survivors were detected and correlated to clinical parameter.

**Methods:**

OHCA survivors with sustained unconsciousness after return of spontaneous circulation (ROSC) were included. PAXgene whole blood samples were drawn immediately after initiation of CPR and subsequently after 6 h, 12 h, 24 h, 48 h, and 72 h. TNF-alpha, IL-8, IL-10, and IL-1ra mRNA levels were quantified by RT-qPCR and compared to multiple organ failure, 30-day survival, and the induction of therapeutic hypothermia (TH).

**Results:**

25 patients (63 ± 15 years) were enrolled presenting a characteristic time-dependent cytokine profile in the early postresuscitation period. High initial TNF-alpha and IL-8 mRNA levels were followed by a significant decrease. IL-1ra mRNA levels significantly increased beginning after 6 h. Nonsurvivors showed significantly higher IL-8 mRNA levels immediately after CPR. TH induced significantly higher IL-1ra mRNA levels compared to normothermia.

**Conclusion:**

Significant mRNA cytokine expression changes are already detectable immediately after initiation of CPR. These expressional changes are significantly different depending on 30-day survival. TH seems to attenuate proinflammatory immune reaction by a significant increase of IL-1ra mRNA levels. This trial is registered with DRKS00012940.

## 1. Introduction

Survival of out-of-hospital cardiac arrest (OHCA) is affected by global ischaemia followed by a pronounced reperfusion injury due to return of spontaneous circulation (ROSC). The ischaemia-reperfusion response induces a complex activation of immunologic pathways leading to subsequent systemic inflammatory immune reaction. This phenomenon is known as the postcardiac arrest syndrome (PCAS). The underlying pathophysiological changes are known to be characterized by a fundamental dysregulation of both, pro- and anti-inflammatory cytokines, including IL-6, IL-8, TNF-alpha, IL-1ra, and IL-10 [1]. The duration of whole-body ischaemia and the subsequent inflammatory release have been identified to influence the severity of PCAS [2, 3]. Furthermore, the extent of these cytokine disorders seems to be associated with postresuscitation outcome [4]. To our knowledge, the majority of clinical studies evaluating cytokine changes started blood sampling when patients arrived at the hospital or were admitted to the ICU. One major interest of this study was to already detect very early expressional cytokine changes. Therefore, blood samples were drawn immediately after initiation of CPR in the preclinical setting in order to elucidate very early functional changes of the human immune system via investigating mRNA expression profiles of selected immunomodulatory genes directly following cardiac arrest. This study further correlated cytokine mRNA expression changes with clinical outcome parameters such as multiple organ failure and 30-day mortality. Secondly, we evaluated the impact of therapeutic hypothermia on cytokine expression profiles as therapeutic hypothermia (TH) has—on the one hand—been shown to beneficially influence mortality rates after cardiac arrest (CA) [5–12]. However, data concerning the impact of hypothermia on the postresuscitation inflammation and the underlying mechanisms of PCAS-related cytokine changes is still conflicting [13–15].

## 2. Methods

### 2.1. Study Setting and Population

Patients (≥18 years) successfully resuscitated from OHCA with sustained unconsciousness (GCS < 8) after ROSC were included between January 2003 and March 2006 after approval of Regional Ethical Review Board of the Ludwig-Maximilians University, Munich, Germany (decision: 282/01). Written informed consent was obtained from each patient's next of kin and/or from patients regaining consciousness after cardiac arrest. The study was performed in accordance with the Declaration of Helsinki and its amendments and European Union guidelines for good clinical practice. Main exclusion criteria were acute infection, intracerebral hemorrhage, aortic dissection, or pregnancy. Prehospital data regarding the cardiac arrest including initial heart rhythm, witnessed collapse, bystander CPR, and time to ROSC were collected according to Utstein guidelines [16, 17]. Further, clinical parameters and comorbidities, cause of cardiac arrest, TH treatment, presence of cardiogenic shock, use of intra-aortic balloon pump (IABP), and the duration of stay in the intensive care unit (ICU) and in the hospital were recorded for each patient. This study was designed to (1) analyze very early mRNA expression profiles of TNF-alpha, IL-8, IL-10, and IL-1ra beginning already after CPR and within the first 72 h after successful cardiopulmonary resuscitation, (2) correlate cytokine levels with clinical outcome parameters, and (3) evaluate the effect of TH on cytokine mRNA expression levels. *Multiple organ failure* (*MOF*) was detected using MOF score as described by Goris et al. and modified by Lefering et al. [18, 19]. A MOF score ≥ 4 was surmised as multiple organ failure. If clinical data sets were incomplete, patients were excluded from this subanalysis. *Survival* was defined as 30-day survival after cardiac arrest. Patients who did not survive the first 24 h after resuscitation were excluded. Therapeutic hypothermia (TH) was not yet implemented in Resuscitation Guidelines at the time of sample collection and therefore not induced regularly. Our patients were treated in eight different hospitals, and a standardized TH protocol was therefore unavailable. In order to reduce heterogeneity, patients were retrospectively divided into two subgroups. Only patients treated according to the same TH protocol were included into the hypothermia group. Hypothermia was achieved by external cooling methods (blanket, ice packs) and associated with routine use of neuromuscular blocking agents. Targeted levels of hypothermia were 32–34°C and maintained for 24 h.

### 2.2. Patient Management

Survivors of OHCA were admitted to one of the eight intensive care units in Munich. All patients received intensive care treatment according to the current guidelines including mechanical ventilation, fluid substitution, antibiotic therapy, SIRS/sepsis management, and vasopressor treatment. Patients were sedated and received adequate analgesia according to the standard of each ICU. Percutaneous coronary intervention was performed if necessary.

### 2.3. Blood Sampling

Blood samples were drawn immediately after initiation of cardiopulmonary resuscitation by one of our ambulance emergency physicians (central ambulance station/LMU Munich) and subsequently 6, 12, 24, 48, and 72 h by one of the study's investigators.

### 2.4. RNA Extraction

Blood was collected in PAXgene tubes, and total RNA was extracted using the *PAXgene Blood RNA Kit* (PreAnalytix, Hombrechtikon, Swiss) according to the manufacturer's instructions. RNA quantity was measured with Nanodrop spectrophotometer ND-1000 (Thermo Scientific).

### 2.5. cDNA Synthesis

An aliquot of 8.2 *μ*l standardized to 1 *μ*g of RNA was reverse transcribed with avian myeloblastosis virus—reverse transcriptase (AMV-RT) and oligo-p(dT)15-primer—following manufacturer's instruction (1st Strand cDNA Synthesis Kit, Roche, Mannheim, Germany). After denaturation (Thermocycler, 65°C for 15 min), samples were iced for 5 minutes and incubated with manufacture's master-mix (11.8 *μ*l including MgCl2, deoxynucleotide-mix, oligo-p(dT)15-primer, AMV reverse transcriptase, and RNase inhibitor).

### 2.6. Quantitative Real-Time PCR (RT-qPCR)

Quantitative real-time PCR was performed in this study. The obtained cDNA was diluted 1 : 25 with water and 10 *μ*l were used for amplification. The quantitative analysis of target gene expression was performed on a LightCycler by real-time PCR using the Light Cycler Fast Start DNA Master Sybr Green I Kit according to the manufacturer's instructions (Roche, Mannheim, Germany). Standard and primer were designed by *Search LC* (Heidelberg, Germany; Light Cycler Primer Sets); RT-qPCR was performed by denaturation and polymerase activation (95°C for 10 min), amplification of RT-qPCR products in a 45 cycle one-step PCR including denaturation (95°C, 10 s)/annealing (68°C–58°C, −0.5°C/cycle)/extension (72°C, 16 s) for each circle. The results of negative control samples were set as baseline level. Results of RT-qPCR were standardized to this baseline level and given as copies/*μ*lRNA. Specificity of the amplification products was verified by melting curve analysis combined with agarose gel electrophoresis. For calculation, data processing and documentation of the LightCycler analysis software version 3.39 (Roche Diagnostics, Germany) was used.

### 2.7. Statistical Analysis

Data were statistically analyzed using ANOVA analysis (*Kruskal-Wallis*) and post hoc Dunn's Test in order to evaluate significant differences regarding the time points (0 h (A), 6 h, 12 h, 24 h, 48 h, and 72 h). Subgroups were tested using ANOVA and post hoc nonparametric Mann–Whitney rank sum test. The results were declared as mean values with standard error of the mean (SEM) and were considered statistically significant for *p* < 0.05. Statistical analysis was performed using SigmaStat (version 4.0)/SigmaPlot (version 11.0).

## 3. Results

### 3.1. Clinical Baseline Characteristics

Twenty-five patients (63 ± 15 years; range: 35–90 years) who were resuscitated from OHCA were included (Table 1). MOF score was calculated in 15 patients detecting multiple organ failure in 60% (MOF < 4: *n* = 6; MOF ≥ 4: *n* = 9). 60% survived after cardiac arrest (survivors *n* = 15; nonsurvivors *n* = 10). TH was induced in six patients following a standardized protocol; seven patients were not admitted to any kind of TH treatment (normothermia: *n* = 7; hypothermia: *n* = 6). Detailed information regarding patient characteristics, cardiac arrest, and postresuscitation treatment are depicted in Table 2.

### 3.2. Time-Course of mRNA Expression Profiles

**Figure 1:**
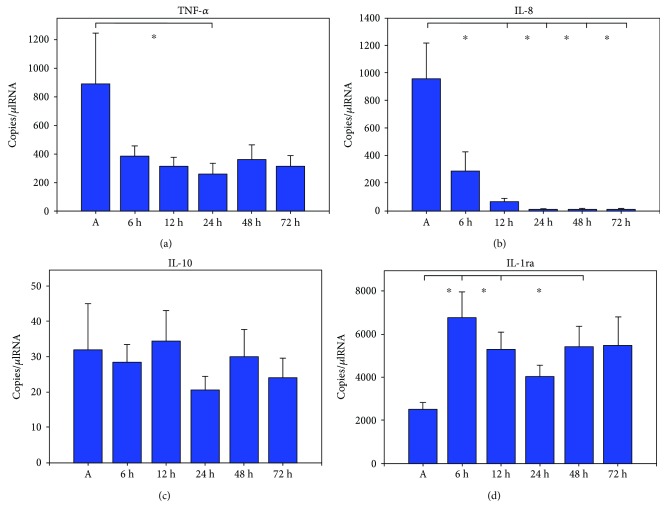
Plots of cytokine mRNA expression of TNF-alpha (a), IL-8 (b), IL-10 (c), and IL-1ra (d) 0 h–72 h after ROSC; bars represent mean values with error bars representing SEM. Kruskal-Wallis test and post hoc Dunn's test ^∗^*p* < 0.05; all patients *n* = 25.

Gene expression dynamics of TNF-alpha, IL-8, IL-10, and IL-1ra compared to mRNA levels immediately after ROSC (A) are illustrated in Figure 1. Values are given in copies/*μ*lRNA.

### 3.3. Proinflammatory Cytokines

Initially after cardiopulmonary resuscitation, TNF-alpha mRNA expression showed very high levels followed by continuous decrease during the course of time with a significant minimum after 24 h (889 ± 335 (A) versus 257 ± 74 (24 h); *p* = 0.002). IL-8 mRNA expression levels showed a peak immediately after cardiac arrest followed by a significant decrease with minimal levels after 12–72 h (1351 ± 489 (A) versus 71 ± 19 (12 h), 1351 ± 489 (A) versus 12 ± 2 (24 h), 1351 ± 489 (A) versus 13 ± 3 (48 h), and 1351 ± 489 (A) versus 12 ± 3 (72 h); *p* < 0.001).

### 3.4. Anti-Inflammatory Cytokines

IL-10 mRNA expression levels showed undulant low values with a gradual nonsignificant increase after 12 h followed by a nonsignificant decrease. IL-1ra mRNA expression levels started on a lower level immediately after CPR followed by a significant increase starting after 6 h (2629 ± 335 (A) versus 6940 ± 1055 (6 h), *p* < 0.001, 2629 ± 335 (A) versus 6144 ± 1055 (12 h), *p* = 0.002, and 2629 ± 335 (A) versus 5900 ± 948 (48 h); *p* = 0.005).

### 3.5. Gene Expression Levels and Clinical Outcome

**Figure 2:**
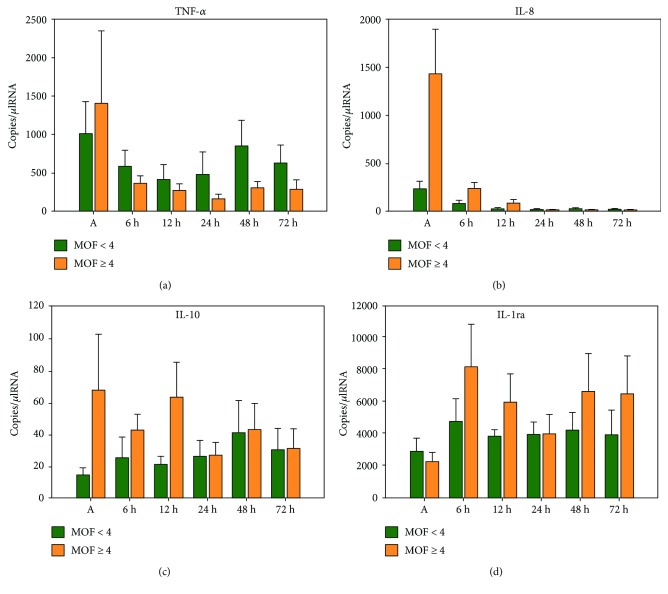
Plots of comparison between mRNA levels of TNF-alpha (a), IL-8 (b), IL-10 (c), and IL-1ra (d) and multiple organ failure assessed by multiple organ failure score (MOF score) 0 h–72 h after cardiac arrest; bars represent mean values with error bars representing SEM. ANOVA and post hoc Mann–Whitney rank sum test; MOF < 4 *n* = 6; MOF ≥ 4 *n* = 9.

**Figure 3:**
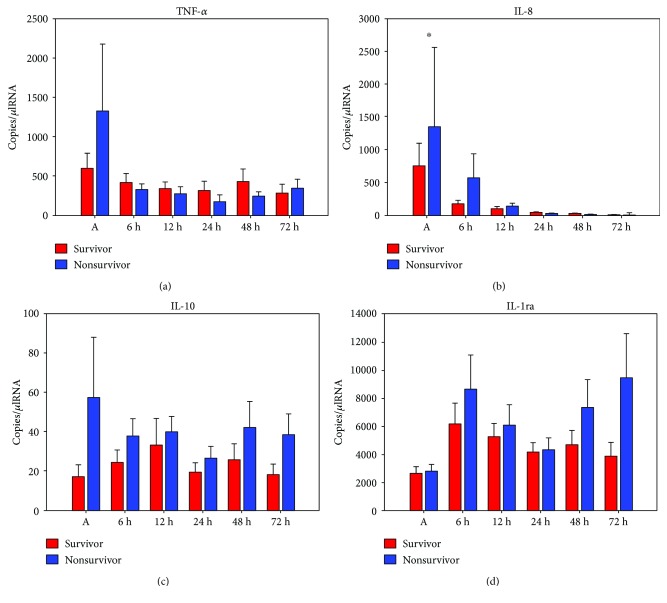
Plots of comparison between mRNA levels of TNF-alpha (a), IL-8 (b), IL-10 (c), and IL-1ra (d) and 30-day survival 0 h–72 h after cardiac arrest; bars represent mean values with error bars representing SEM. ANOVA and post hoc Mann–Whitney rank sum test ^∗^*p* < 0.05; survivors (*n* = 15) and nonsurvivors (*n* = 10).

IL-8, IL-10, and IL-1ra mRNA levels were higher in patients suffering from multiple organ failure without revealing statistically significant differences (Figure 2). Nonsurvivors expressed significantly higher IL-8 mRNA levels immediately after initiation of CPR when compared to survivors (719 ± 349 (survivors) versus 2300 ± 1157 (nonsurvivors); *p* = 0.02; Figure 3). IL-10 mRNA levels were significantly elevated after 48 h (23 ± 8 (survivors) versus 40 ± 13 (nonsurvivors); *p* = 0.046) and 72 h (16 ± 5 (survivors) versus 37 ± 9 (nonsurvivors); *p* = 0.034) in nonsurvivors. TNF-alpha and IL-1ra mRNA levels did not show any significant differences between the two groups.

### 3.6. Gene Expression Levels and Therapeutic Hypothermia

**Figure 4:**
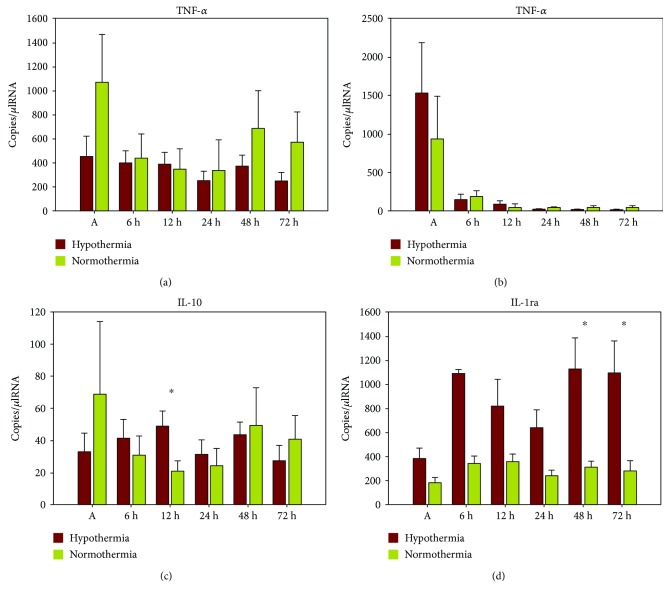
Plots of comparison between mRNA levels of TNF-alpha (a), IL-8 (b), IL-10 (c), and IL-1ra (d) and therapeutic hypothermia 0 h–72 h after cardiac arrest; bars represent mean values with error bars representing SEM. ANOVA and post hoc Mann–Whitney rank sum test ^∗^*p* < 0.05; hypothermia (*n* = 6); normothermia (*n* = 7).

IL-1ra mRNA expression levels were higher in all patients following hypothermia, reaching significant higher levels after 48 h (3369 ± 640 (normothermia) versus 9629 ± 2330 (hypothermia); *p* = 0.019) and 72 h (3588 ± 1040 (normothermia) versus 10,886 ± 2183 (hypothermia); *p* = 0.014) in the hypothermia group (Figure 4). Patients treated with TH showed significantly higher IL-10 mRNA levels after 12 h when compared to normothermia group (20 ± 6 (normothermia) versus 48 ± 9 (hypothermia); *p* = 0.028). TNF-alpha and IL-8 mRNA levels did not show any significant changes.

## 4. Discussion

To our knowledge, this is the first study evaluating mRNA expression changes starting immediately after initiation of cardiopulmonary resuscitation. Via detecting very early mRNA expression changes of both pro- and anti-inflammatory cytokines, we could demonstrate a characteristic gene expression kinetic following cardiac arrest. Furthermore, we could detect significant cytokine alterations depending on clinical outcome directly after cardiac arrest. Therapeutic hypothermia leads to significant higher IL1-ra mRNA levels when compared to patients treated with normothermia.

### 4.1. Mediators of Interest

Several cytokines have already been identified to play a pivotal role in postresuscitation immunological changes and have therefore been chosen for the present investigation. TNF-alpha and interleukin-8 have a high amplifying potential regarding the primary systemic response after ROSC [2, 4, 20, 21]. Interleukin-10 is known as an essential PCAS-related anti-inflammatory cytokine controlling the downregulation of proinflammatory cytokines [13]. Anti-inflammatory IL-1ra has been focused in postresuscitation studies functioning as a marker of IL-1 activity [22, 23].

### 4.2. Results

The highlight of this investigation is the possibility to demonstrate such early mRNA expression changes in this vulnerable patient population following cardiopulmonary resuscitation. Thereby, we could show that TNF-alpha and IL-8 mRNA levels were expressively high immediately after cardiac arrest followed by a significant decrease over the next 72 hours. These results confirm previous findings demonstrating a rapid upregulation of proinflammatory markers after ROSC. In this context, Adrie et al. and Ito et al. reported similar time-course changes of TNF-alpha and IL-8 in their cohorts showing a very early cytokine release after the ischemic event [20, 24]. However, in contrast to our study, both groups only started their observations when patients were admitted to the hospital/intensive care unit. In their experimental setting, Niemann et al. could detect early changes showing increased TNF-alpha levels within 30 minutes after ROSC [25]. Regarding the characteristic cytokine release, our observations are in line with Adrie et al. who also documented a subsequent decrease of TNF-alpha and IL-8 in the later course of the following three days [24]. Bro-Jeppesen et al. also found high initial TNF-alpha concentrations followed by a significant decrease within the first 72 hours after ROSC [3]. On the anti-inflammatory side, IL-10 expression was constantly low in our cohort whereas IL-1ra levels started on a lower baseline level and increased significantly beginning 6 h after ROSC. In summary, cytokine mRNA expression underlie an immediate characteristic time-course in our cohort showing a significant downregulation of pro- and upregulation of anti-inflammatory cytokines reflecting the coexistence of both in case of PCAS. This might be understood as an attempt of the postresuscitation organism to attenuate the progressive inflammation mediated by proinflammatory cytokines by raising the anti-inflammatory immune defense [26]. In contrast to numerous studies that investigate cytokine changes in serum or plasma, a major strength of this work is the evaluation of mRNA expression changes. This is based on the idea that changes on genome level can improve the understanding of underlying immune functionality. To our knowledge, there are solely some experimental studies available that evaluate mRNA expression levels after cardiac arrest. Meybohm et al. demonstrated a similar upregulation of TNF-alpha, ICAM-1, and IL-1b mRNA levels in cerebral tissue [14]. In contrast, Sipos et al. interpreted the downregulation of TNF-alpha and IL-2 mRNA levels in their study as an immune paralyze after cardiac arrest [1]. Major strength of these two studies is the evaluation of both mRNA and protein levels. The missing protein analysis might thereby be seen as a limitation of our work as mRNA levels do not necessarily correlate with protein levels. Particularly, IL-10 is known to underlie posttranscriptional regulation processes articulately influencing later protein levels. This might explain the diverse data concerning IL-10 expression levels in our series. However, one of the main advantages in studying gene expression profiles is the detection of very early transcriptome changes. By evaluating these changes starting in a preclinical setting, we could demonstrate that these fundamental functional immunological changes already start immediately after cardiac arrest.

### 4.3. Outcome

Several groups elucidated the correlation of PCAS-related cytokine changes and clinical outcome. Our results are in line with previous findings as we could detect a significant upregulation of IL-8 mRNA levels in nonsurvivors versus survivors immediately after cardiac arrest [20]. This phenomenon is only detectable in the very early postresuscitation period followed by a downregulation of IL-8 cytokine expression in both survivors and nonsurvivors. Similarly, TNF-alpha showed higher initial mRNA levels in nonsurvivors but did not reveal statistical significance. Whereas the proinflammatory immune reaction started immediately after cardiac arrest in our study, anti-inflammatory cytokine dynamics started minimally temporally delayed demonstrating significant higher IL-10 mRNA levels 48 h and 72 h after the beginning of CPR in nonsurvivors. However, IL-10 mRNA levels were very low in our cohort during the whole observation period. Therefore, the value of these results remains questionable. Peberdy et al. recently documented significant higher IL-1ra, IL-6, IL-8, and IL-10 levels in nonsurvivors and patients with poor functional outcome which is similar to our results showing higher IL-1ra expression levels in nonsurvivors [21]. However, IL-1ra levels did not reveal statistical significance presumably on account of the small sample size. With regard to the devolvement of multiple organ failure, no statistical significance could be detected between the two groups. By trend, patients suffering from multiple organ failure showed higher mRNA levels of IL-8, IL-10, and IL-1ra.

### 4.4. Impact of Therapeutic Hypothermia

By studying early cytokine changes after cardiac arrest, we could further show a significant increase of anti-inflammatory IL-1ra mRNA levels in patients treated with TH. Simultaneously, proinflammatory TNF-alpha mRNA levels were lower in the hypothermia group by trend without revealing statistical significance. From our point, these findings might support the relationship between postresuscitation inflammatory parameters and induced hypothermia. This temperature-related effect might be interpreted as a beneficial impact of TH on cytokine dynamics that attenuates the proinflammatory “injury” by raising anti-inflammatory mechanisms. Major strength of this study is the normothermic control group as the study was conducted when TH was not yet implemented in the guidelines. Based on several studies, hypothermia is strongly advocated in postresuscitation patients to reduce PCAS-related neurologic dysfunction and improve outcome [5–7]. Nevertheless, the impact on the enhanced systemic immune reaction has not been fully understood, as available data is still conflicting. Meybohm et al. demonstrated a significant reduction of elevated proinflammatory cytokines due to hypothermia [14]. They concluded a positive influence on outcome via reduced apoptosis rates and proinflammatory cytokine expression. Similar inhibitory effects of TH represented by a decrease of inflammatory response have been stated by Yanagawa et al. [27]. In their study, the ischemic brain incubated at 33°C showed decreased IL-6 levels when compared to 36°C. Contrary, Sipos et al. could not demonstrate any effect of TH on cytokine expressions [1]. Regarding the available clinical studies, results and conclusions are widely diverging. Fries et al. demonstrated a suppressed release of CRP but not IL-6 by induction of TH [13]. Aibiki et al. have demonstrated a decreased proinflammatory immune reaction and improved outcome as a result of TH [28]. In contrast, Bisschops et al. concluded that hypothermia could not prevent high proinflammatory cytokine levels [29]. Similarly, Callaway et al. did not find any association between cytokine changes and neurologic recovery after hypothermia and concluded that damping the inflammatory response might not be the reason for the beneficial effect of TH [30]. Bro-Jeppesen et al. demonstrated, in their randomized controlled trial, that mediators of the pro- and anti-inflammatory response were not significantly influenced by TTM at 33°C compared to 36°C [15]. When evaluating this conflicting data, the majority of publications suggest that the benefit of hypothermia is the “pan inhibition” of ischaemia-induced damaging pathways. However, Zhao et al. recently emphasized another potential effectiveness of TH. They hypothesized that the beneficial impact might result from the induction of protective genes and not only from a down streaming of detrimental ones [31]. Referring to this, Hicks and colleagues found that experimentally induced hypothermia was associated with distinct changes of stress-induced protein expressions [8]. Regarding our results, we therefore conclude that TH reduces proinflammatory mRNA cytokine expression as seen by lower TNF-alpha levels and a significant upregulation of IL-1ra expression, which becomes especially remarkable after 6–72 h, potentially representing a protective anti-inflammatory immune response. Nevertheless, the positive effect of TH via modification of anti-inflammatory cytokine expression profiles remains unclear and might therefore only serve as an attempt to explain our results. As IL-1ra mRNA levels were—by tendency—also higher in nonsurvivors and patients suffering from MOF, TH might not be the only reason for the anti-inflammatory response. Due to the small sample size, further subgroup analysis (survivors versus nonsurvivors with or without TH) was not possible.

### 4.5. Limitations of This Study

Main limitations of our work are the small sample size and heterogeneous cohort due to the challenging clinical setting. Our emergency physician collected the first blood sample preclinical immediately after initiation of CPR in order to evaluate cytokine changes as early as possible. Afterwards, patients were admitted to various hospitals in town leading to nonstandardized treatment and difficulties in completing all data sets. The induction of TH varied significantly in the different hospitals. In order to reduce heterogeneity in this subcohort, only patients who were treated according to the same protocol were included in TH group leading to small subgroups.

## 5. Conclusion

The very early postcardiac arrest interval is characterized by a significant pro- and anti-inflammatory cytokine expression time-course. Nonsurvivors express significantly higher IL-8 mRNA levels immediately after cardiac arrest. Following successful resuscitation, therapeutic hypothermia is accompanied by a significant upregulation of IL-1ra mRNA potentially suggesting a protective effect of TH by raising the anti-inflammatory postresuscitation immune defense.

## Figures and Tables

**Table 1:** Patient characteristics.

Pt.	Gender (m/f)	Age (year)	Diagnosis Initial heart rhythm	Number of shocks	Hypothermia	Outcome
1	m	62	Respiratory failure (COPD) Asystole	—	U	S
2	m	76	Myocardial infarction Ventricular fibrillation	6	U	S
3	m	63	Respiratory failure (COPD) Ventricular fibrillation	1	U	NS
4	m	32	Asphyxia Asystole	—	U	NS
5	m	34	Chest trauma Asystole	—	U	S
6	m	61	Myocardial infarction Asystole	—	U	S
7	m	86	Myocardial infarction Asystole	—	U	NS
8	m	56	Myocardial infarction Ventricular fibrillation	3	U	S
9	m	45	Myocardial infarction Ventricular fibrillation	3	U	S
10	m	44	Myocardial infarction Ventricular fibrillation	5	U	S
11	m	67	Myocardial infarction Asystole	—	U	S
12	f	72	Lung embolism Asystole	—	U	NS
13	m	54	Unknown Ventricular fibrillation	3	N	NS
14	m	61	Myocardial infarction Ventricular fibrillation	6	N	S
15	m	90	Cardiac arrhythmia Ventricular fibrillation	1	N	S
16	m	51	Myocardial infarction Ventricular fibrillation	1	N	S
17	f	62	Coronary heart disease Ventricular fibrillation	9	N	NS
18	m	70	Coronary heart disease Ventricular fibrillation	1	N	S
19	f	62	Coronary heart disease Ventricular fibrillation	2	N	S
20	f	51	Myocardial infarction Ventricular fibrillation	7	Y	NS
21	f	69	Myocardial infarction Ventricular fibrillation	10	Y	S
22	m	72	Myocardial infarction Asystole	—	Y	NS
23	m	88	Myocardial infarction Ventricular fibrillation	4	Y	NS
24	f	82	Respiratory failure (COPD) Ventricular fibrillation	1	Y	NS
25	m	75	Coronary heart disease Ventricular fibrillation	1	Y	S

N: no; NS: nonsurvivor; S: survivor; U: unclear; Y: yes.

**Table 2:** Patient characteristics.

Patient characteristics	Number (no.)/value (mean ± SD; range)
*Patient factors*	
Age	63 ± 15 (35–90 years)
Gender (m/f)	19/6
Outcome (survivors/nonsurvivors)	15/10
*Cause of cardiac arrest*	
Cardiac disease	18
Respiratory failure	3
Lung embolism	1
Asphyxia	1
Trauma	1
Unknown	1
*Initial cardiac rhythm*	
Ventricular fibrillation	17
Asystole	8
*Event factors*	
Witnessed collapse	9
Bystander CPR	8
Time to ROSC in minutes	21 ± 13 (5–50 minutes)
Number of shocks (*n* = 17)	4 ± 3 (1–10)
*Treatment*	
Hypothermia/normothermia	6/7
